# Avian Bornavirus Research—A Comprehensive Review

**DOI:** 10.3390/v14071513

**Published:** 2022-07-11

**Authors:** Dennis Rubbenstroth

**Affiliations:** Institute of Diagnostic Virology, Friedrich-Loeffler-Institut, 17493 Greifswald, Insel Riems, Germany; dennis.rubbenstroth@fli.de

**Keywords:** avian bornaviruses, *Bornaviridae*, orthobornaviruses, proventricular dilatation disease (PDD), parrots, Psittaciformes, pathogenesis, epidemiology, diagnostic strategies, vaccines

## Abstract

Avian bornaviruses constitute a genetically diverse group of at least 15 viruses belonging to the genus *Orthobornavirus* within the family *Bornaviridae*. After the discovery of the first avian bornaviruses in diseased psittacines in 2008, further viruses have been detected in passerines and aquatic birds. Parrot bornaviruses (PaBVs) possess the highest veterinary relevance amongst the avian bornaviruses as the causative agents of proventricular dilatation disease (PDD). PDD is a chronic and often fatal disease that may engulf a broad range of clinical presentations, typically including neurologic signs as well as impaired gastrointestinal motility, leading to proventricular dilatation. It occurs worldwide in captive psittacine populations and threatens private bird collections, zoological gardens and rehabilitation projects of endangered species. In contrast, only little is known about the pathogenic roles of passerine and waterbird bornaviruses. This comprehensive review summarizes the current knowledge on avian bornavirus infections, including their taxonomy, pathogenesis of associated diseases, epidemiology, diagnostic strategies and recent developments on prophylactic and therapeutic countermeasures.

## 1. Introduction

Avian bornaviruses were discovered in 2008 in parrots suffering from proventricular dilatation disease (PDD) [1,2]. PDD is a chronic neurologic and intestinal disorder of psittacine birds (order Psittaciformes) that was first described in the late 1970s in Europe and the USA. Since initially mainly macaws appeared to be affected, the disease was first described as ‘macaw wasting disease’. Additional synonyms included ‘neurotropic gastric dilatation’, ‘myenteric ganglioneuritis’ and ‘infiltrative splanchnic neuropathy’ [3,4,5,6]. However, PDD was soon found to occur in captive individuals of more than 70 psittacine species on several continents [3,4,5]. Diseases resembling PDD were sporadically detected also in non-psittacine species [5,7,8].

Although its etiology remained unknown for three decades, a transmissible nature of PDD was soon suspected based on field observations and experimental induction of the disease by transfer of tissue homogenate from diseased birds to healthy individuals [4,6]. The typical occurrence of non-suppurative encephalitis and ganglioneuritis with mononuclear infiltrates pointed towards a viral origin [3,5,9,10,11,12]. Several viruses had been discussed as possible causative agents of PDD, including paramyxoviruses, coronaviruses, alphaviruses and adenoviruses, but evidence was lacking [5,6,9]. Finally, in 2008, a group of avian bornaviruses was discovered in association with PDD [1,2], and Henle-Koch’s postulates were fulfilled by experimental reproduction of the disease [13,14,15,16,17].

Subsequently, additional genetically diverse avian bornaviruses were discovered not only in psittacines but also in birds of the orders Passeriformes, Anseriformes and Charadriiformes [18,19,20,21,22,23,24,25,26].

## 2. Taxonomy and Nomenclature of the Family *Bornaviridae*

Bornaviruses are members of the family *Bornaviridae* within the order *Mononegavirales.* Currently, the family comprises the three genera *Orthobornavirus*, *Carbovirus* and *Cultervirus* [27,28].

Due to the discovery of various new bornaviruses during the past 15 years, the taxonomy and nomenclature of the family underwent several updates [27,28,29,30,31,32], which is likely to continue in the future. Only recently, the International Committee on Taxonomy of Viruses (ICTV) has introduced a more uniform format for viral species names, which now have to follow a binominal Linnean format [33]. These new rules were also implemented for the family *Bornaviridae* [28], and the new species names are used throughout this review article (Table 1).

### 2.1. Avian Bornaviruses

All bornaviruses of avian origin known to date belong to the genus *Orthobornavirus*. Following their first discovery, they had been tentatively described as different genotypes of a single virus named ‘avian bornavirus’ (ABV). However, when it became clear that avian bornaviruses did not represent a single monophyletic and genetically uniform virus but constituted a heterogenous group of independent viruses with a broad genetic variability, the virus name ‘avian bornavirus’ was abandoned and its former genotypes were classified as currently 15 separate viruses within five different viral species (Table 1) [27,28].

The bornaviruses of Psittaciformes, namely parrot bornavirus 1 to 8 (PaBV-1 to PaBV-8), are genetically highly divergent and belong to two separate viral species, *Orthobornavirus alphapsittaciforme* (PaBV-1 to 4, PaBV-7 and -8) and *Orthobornavirus betapsittaciforme* (PaBV-5 and -6) (Table 1) [24,27,28]. Additional avian bornaviruses have been discovered in passerine birds (order Passeriformes) and in aquatic birds of the orders Anseriformes and Charadriiformes. The passerine bornaviruses belong to the viral species *Orthobornavirus serini* (canary bornavirus 1 to 3 (CnBV-1 to CnBV-3) and munia bornavirus 1 (MuBV-1)) and *Orthobornavirus estrildidae* (estrildid finch bornavirus 1 (EsBV-1)). Aquatic bird bornavirus 1 and 2 (ABBV-1 and ABBV-2) form the species *Orthobornavirus avisaquaticae* [27,28].

### 2.2. Non-Avian Bornaviruses

**Figure 1:**
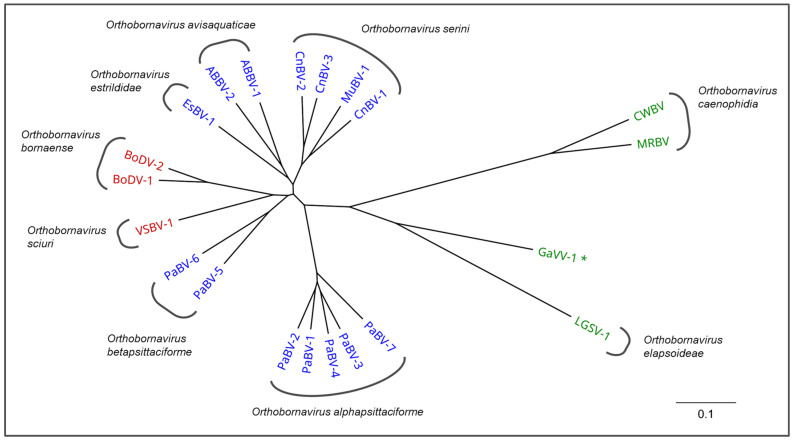
Phylogeny of the genus *Orthobornavirus*. Complete P gene sequences (606 nucleotides) of representative bornavirus sequences from birds (blue), mammals (red) and reptiles (green) were analyzed using neighbor-joining algorithm and Jukes–Cantor distance model in Geneious R11 software. Values at branches represent support in 1000 bootstrap replicates. Only bootstrap values ≥ 70 at major branches are shown. * GaVV-1 has not been classified by the International Committee on Taxonomy of Viruses (ICTV) yet. ABBV: aquatic bird bornavirus, BoDV: Borna disease virus, CnBV: canary bornavirus, EsBV: estrildid finch bornavirus, GaVV: Gabon viper virus, LGSV: Loveridge´s garter snake virus, MuBV: munia bornavirus, PaBV: parrot bornavirus, VSBV: variegated squirrel bornavirus.

The mammalian orthobornaviruses Borna disease virus 1 and 2 (BoDV-1 and -2; both species *Orthobornavirus bornaense*) and variegated squirrel bornavirus 1 (VSBV-1; species *Orthobornavirus sciuri*) are the closest relatives of the avian bornaviruses (Figure 1). The well-described BoDV-1 is the causative agent of Borna disease, a neurologic disorder of horses, sheep, humans and other mammals in central Europe [27,34,35]. VSBV-1 was recently discovered in captive squirrels in Europe and described to cause lethal zoonotic infections in humans [32,36,37,38].

Several more distantly related reptilian orthobornaviruses have been described exclusively in snakes [39,40,41]. Relatively little is known about the genera *Carbovirus* and *Cultervirus* that comprise viruses of snakes or fish, respectively [28,32,42,43].

## 3. Orthobornavirus Genome Structure and Proteins

### 3.1. Genome Organization

**Figure 2:**
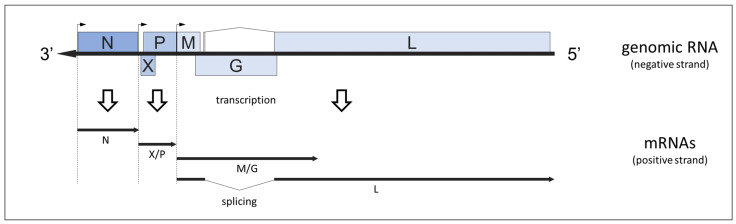
Organization of the *Orthobornavirus* genome. The negative-sense genome (presented in 3′-to-5′ orientation) of orthobornaviruses contains open-reading frames (ORF) encoding six major proteins: N = nucleoprotein, X = accessory protein X, P = phosphoprotein, M = matrix protein, G = glycoprotein, L = large RNA-dependent RNA polymerase. The structure is based on the BoDV-1 reference genome (GenBank accession number NC_028100). ORFs shown in identical shadings of blue are translated from the same bi- or polycistronic mRNA. Major mRNA variants are shown as black arrows in 5′-to-3′ orientation below the genome.

Bornaviruses possess a monopartite single-stranded RNA genome of negative polarity and approximately 9000 nucleotides (nt) length. Orthobornavirus genomes encode for six proteins: nucleoprotein (N), accessory protein p10 (X), phosphoprotein (P), matrix protein (M), glycoprotein (G) and the large RNA-dependent RNA polymerase (L) (Figure 2) [28,44,45,46], while carboviruses and culterviruses have slightly different genome structures [28,42,43].

At least four polyadenylated and capped messenger RNAs (mRNAs) are transcribed from the orthobornavirus genome. A monocistronic mRNA encodes for the N protein, whereas the overlapping X and P open reading frames (ORFs) are located together on a bicistronic mRNA (Figure 1) [28,45]. Two further polycistronic mRNAs of 2.8 or 7.2 kilo bases length are covering the M, G and L ORFs (Figure 1), with the longer transcript being the result of transcriptional read through at a transcription termination site following the G ORF. To translate either M, G or L protein from these transcripts, orthobornaviruses employ mRNA editing by the cellular splicing machinery [28,45,47].

A small ORF of 30 nt length located upstream of the X ORF (uORF) was shown to be involved in the regulation of X and P protein expression of BoDV-1. Since the uORF overlaps with the X ORF, it negatively affects the initiation of X ORF translation and thereby facilitates ribosomal re-initiation at the P ORF [48,49]. The intergenic region between the N and the X gene is highly variable among the avian bornavirus genomes. While some viruses possess uORFs of different lengths, it is completely absent in the genomes of others [19,20]. The biological relevance of these genomic variations remains unknown.

### 3.2. Function of Gene Products

The functions of the orthobornavirus proteins have been studied mainly for BoDV-1 and rudimentarily for other orthobornaviruses, including avian bornaviruses.

The N protein packages the viral RNA and is thereby part of the ribonucleoprotein (RNP) complex and essential for viral polymerase activity [50,51,52]. It possesses a nuclear localization signal (NLS) as well as a nuclear export signal (NES), suggesting that it is involved in shuttling RNPs into and out of the nucleus [45,53,54].

The P protein is phosphorylated by cellular kinases and its NLS directs it into the nucleus. As an essential co-factor of the bornavirus polymerase, it is part of the RNP, and it is able to interact with the N, L and X proteins and with other P molecules [55,56,57,58]. P protein expression is strictly regulated in infected cells as even small variations of the P protein levels may have effects on viral polymerase activity [59].

The accessory protein X is the only orthobornavirus protein not shown to be incorporated in the viral particle [60]. It mainly acts by interacting with the P protein and directing it to the cytoplasm. Since decreased nuclear P protein levels negatively affect polymerase activity, the X protein is considered a negative regulator of bornavirus replication [40,55,61,62].

The M protein is a non-glycosylated protein associated with the inner layer of the viral envelope [45,63]. In infected cells, it co-localizes with protein components of the RNP as well as with viral RNA [64]. Antibodies directed against BoDV-1 M have been described to possess neutralizing activity [65,66].

The G protein is the N-glycosylated surface protein of the bornavirus particle, which is cleaved by cellular furin-like proteases. The full-length isoform (gp94) of the protein mediates viral attachment to a still unknown receptor on the cell surface, whereas the C-terminal cleavage product (gp43) is involved in pH-dependent membrane fusion in the endosomes [67,68,69,70,71]. G protein expression is also required for viral spread via direct cell-to-cell contacts in cell cultures [72]. Anti-G antibodies block viral infection and cell-to-cell spread [66,68,69,70,72]. In the cell, G protein is mainly detected in association with the endoplasmic reticulum [70,73,74]. Surprisingly, G protein expression is detectable only in a minority of BoDV-1-infected cells in cell culture as well as in infected hosts [67,70,71,73,75,76,77]. The same phenotype was observed also for cell cultures infected with avian bornaviruses [75]. The role and the mechanisms of this remarkable regulation of G protein expression are not well understood.

The L protein is by far the largest bornavirus protein and represents the viral RNA-dependent RNA polymerase. The protein is translated from an mRNA resulting from transcriptional read through and splicing [45]. It is a component of the RNP, localizes in the nucleus of infected cells and interacts with the P protein. Phosphorylation by cellular kinases has been shown [78].

### 3.3. Infection and Replication Cycle

It was demonstrated that BoDV-1 particles enter the host cell via receptor-mediated endocytosis triggered by interaction of the G protein with an unknown cellular receptor. After lysosomal pH reduction, the RNP is released into the cytoplasm by G-protein-dependent fusion of viral and cellular membranes [67]. In contrast to most other viruses of the order *Mononegavirales*, the bornavirus RNP is transported into the nucleus, where replication and transcription take place [45,46]. In the nucleus, the virus employs the cellular splicing machinery for alternative splicing of viral mRNAs, thereby regulating expression of its gene products [47].

Bornaviruses establish a non-cytolytic infection, leading to persistence in infected cells by strictly regulating viral replication. The actions of X protein and the N protein isoform p38 as well as a balanced ratio of cellular N and P protein levels are discussed to be involved in this regulatory process [45]. Moreover, X-protein-mediated inhibition of cellular apoptosis is believed to contribute to persistent infection [79] and protect against neuronal degeneration [80].

BoDV-1 particles are released by budding from the cellular wall, but the precise mechanism remains unknown [81]. In general, bornaviruses appear to be strongly cell-associated and only extremely low titers of infectious bornavirus particles are released into supernatants of infected cell cultures [82]. Viral spread appears to occur also directly via cell-to-cell contact, but the mode of transfer remains to be elucidated [45].

## 4. Host Range and Epidemiology of Avian Bornaviruses

### 4.1. Bornaviruses of Psittacines

#### 4.1.1. Captive Psittacine Populations

Avian bornavirus infections have been described in a wide range of families within the order Psittaciformes, and it has to be assumed that the vast majority of psittacine species are susceptible to infection. The viruses can be found in captive psittacine populations worldwide [1,2,21,24,83,84,85,86,87]. Information on the distribution of avian bornaviruses is mainly based on opportunistic sampling of birds presented to veterinary clinicians or submitted to veterinary pathologists, whereas systematic prevalence studies have not been published. In the most extensive study, Heffels-Redmann et al. [83] found 23% of 1442 captive psittacines from several European countries to be positive for markers of avian bornavirus infection (viral RNA detection and/or bornavirus-reactive antibodies), suggesting a wide distribution of these viruses.

Infections with PaBV-2 and PaBV-4 are regularly diagnosed in captive psittacines, with PaBV-4 being the most frequently detected variant [13,18,21,25,83,86,88]. In contrast, all other psittacine bornaviruses known to date, namely PaBV-1, PaBV-3 and PaBV-5 to PaBV-8, have been detected in less than ten birds each [21]. Mixed infections of an individual bird with two bornaviruses, such as PaBV-2 and PaBV-4, PaBV-2 and PaBV-6 or PaBV-4 and PaBV-7, have been described on few occasions [18,88,89]. Associations of bornaviruses or their genetic subclusters with particular psittacine species or geographic regions have not been observed [21]. It is assumed that co-housing of birds of various psittacine species and extensive worldwide trade of those birds have contributed to the wide distribution of the virus.

#### 4.1.2. Free-Ranging Psittacine Populations

Only very limited data are available on avian bornaviruses in free-ranging indigenous or introduced psittacine populations. In the most extensive study, Kessler et al. [90] did not detect avian bornavirus RNA or bornavirus-reactive antibodies in samples from 449 free-ranging ring-necked parakeets from Germany and France. Likewise, Villanueva et al. [91] could not detect bornavirus-reactive antibodies in sera from eight wild parrots from Peru. Encinas-Nagel et al. [92] suspected low levels of bornavirus RNA and bornavirus-reactive antibodies in birds confiscated from smugglers in Brazil. However, most of these birds had been in rehabilitation centers at least for several weeks prior to sampling and may, therefore, not be representative of wild populations. In a study in Australia, seroreactivity was observed in one out of twenty-four sera collected from wild cacatuids, but direct virus detection confirming an infection was not achieved [93]. Thus, evidence for the presence of avian bornaviruses in wild psittacines is still missing.

#### 4.1.3. PaBV Infections in Other Avian Species

Psittacine bornavirus infection of non-psittacine birds has been described for a captive Himalayan monal (*Lophophorus impejanus*). The bird died from neurologic disorders, and PaBV-4 RNA as well as bornavirus antigen were detected in its brain. The bird had been housed together with PaBV-4-infected parrots and viral sequences obtained from the monal and the parrots were almost identical, suggesting the parrots as the source of virus transmission [94]. Reports on the detection of PaBV-2 and PaBV-4 in various wild bird species in Japan require further confirmation [95]. The bornavirus sequences obtained from these wild birds were genetically closely related to sequences from captive psittacines analyzed in the same laboratory. The few observed sequence variations contained an unusually high proportion of non-synonymous mutations, suggesting that these findings may have resulted from laboratory contamination with subsequent sequencing errors [21].

### 4.2. Bornaviruses of Passerines

CnBV-1, CnBV-2 and CnBV-3 have been described to be widely distributed in captive populations of common canaries (*Serinus canaria* forma domestica) in Germany. In two opportunistic surveys, about 20% of the analyzed birds and 40% of all sampled canary holdings tested positive for at least one of these viruses [19,21]. Unlike psittacine bornaviruses, CnBV-1 to CnBV-3 appear to have a rather narrow host range. So far, these viruses have been found exclusively in common canaries but not in other passerine birds, even when housed together with infected canaries [19,20,21,26].

Additional passerine bornaviruses were discovered in members of the family estrildid finches (Estrildidae). Two black-rumped waxbills (*Estrilda troglodytes*) and a yellow-winged pytilia (*Pytilia hypogrammica*) from the same flock in Germany tested positive for EsBV-1 [20]. Sequences of MuBV-1 were identified in sequence database entries originating from white-rumped munias (*Lonchura striata*) that had been analyzed during behavioral studies in Japan [19,96].

With the exception of MuBV-1 in Japan, avian bornaviruses of captive passerines have been described only in central Europe. However, this is likely to be the result of a sampling bias as studies from other parts of the world have not yet been published [19,20,21,26]. None of these viruses were found during surveys of a total of 128 wild passerines collected in Germany [20,21].

### 4.3. Bornaviruses of Aquatic Birds

In contrast to all other known avian bornaviruses, ABBV-1 and ABBV-2 have been found almost exclusively in wild bird populations. ABBV-1 was found in several species of the order Anseriformes, including Canada geese (*Branta canadensis*) and mute swans (*Cygnus olor*) in the USA, Canada, Denmark, Germany and Poland [21,23,97,98,99,100,101,102]. In addition, ABBV-1 infection was reported from birds of the order Charadriiformes, such as a Eurasian oystercatcher (*Haematopus ostralegus*) from Germany [21]. Detection of ‘ABBV-1-like’ viruses in gulls from the USA was described, but sequences allowing identification of the detected virus were not provided [103]. The detection rates were highly variable, ranging from 0.5 to 52% depending on species, sample type and geographic origin [21,23,98,99,100,102]. Considering that North American and European ABBV-1 sequences belong to clearly separated phylogenetic clusters, intercontinental transmission seems rather rare [21,102].

ABBV-2 has been found so far only in a small number of mallards (*Anas platyrhynchos*) and a wood duck (*Aix sponsa*) in North America [22].

Neither virus has been detected in domestic poultry populations thus far. Transmission of ABBV-1 to a non-aquatic bird has been confirmed for a diseased emu (*Dromaius novaehollandiae*) in the Toronto zoo. Canada geese from an ABBV-1-infected resident population, which frequently had been observed grazing in the enclosure in which the emu was kept, are suspected to be the source of infection [104]. Whether such erroneous hosts contribute to the distribution of the virus requires further investigation.

### 4.4. Alleged BoDV-1 Infections in Birds

The mammalian orthobornavirus BoDV-1 is known to be prevalent in wild populations of bicolored white-toothed shrews (*Crocidura leucodon*), from which it can be transmitted to domestic mammals or humans. The virus was shown to be confined to a rather restricted endemic area covering only parts of Germany, Austria, Switzerland and the principality of Liechtenstein [35,105]. In addition to a broad range of mammals, successful experimental BoDV-1 infection was also reported for chickens [106,107], indicating that birds may be susceptible to this virus. However, evidence for natural BoDV-1 infection has not been provided. Surveys of wild and domestic birds in Germany, including samples from the known endemic areas, found no evidence of BoDV-1 infection despite the use of RT-PCR assays that are generally able to detect this virus [18,19,20,21].

Berg et al. [108] reported the detection of BoDV-1-specific RNA in fecal samples of a mallard and a jackdaw (*Corvus monedula*) in Sweden. Retrospective analysis grouped the sequences within a regional BoDV-1 cluster originating from Bavaria to Lower-Saxony [109]. Re-evaluation of the samples in two independent laboratories could not confirm the viral RNA detection, suggesting that the findings of Berg et al. [108] resulted from laboratory contamination [109].

In the 1990s, a series of publications reported the detection of a bornavirus in juvenile ostriches (*Struthio camelus*) suffering from a paretic syndrome in Israel [110,111,112,113,114]. Based on serology and antigenic characterization, the virus was regarded as BoDV-1, which was the only known bornavirus at that time. However, retrospectively, the positive results appear more likely to have been caused by a different serological cross-reactive agent.

## 5. Avian Bornavirus Transmission and Course of Infection

### 5.1. Horizontal Transmission

The routes and mechanisms involved in the transmission of avian bornaviruses are poorly understood. Phylogenetic data suggest horizontal transmission within and between host species to play an important role in the distribution of at least some avian bornaviruses. This conclusion is based on nearly identical viral sequences found in parallel in various species, resulting in a lack of host-associated virus clusters [21]. However, horizontal transmission appears to be rather inefficient in subadult and adult birds since individuals co-housed with persistently infected birds may stay free of detectable virus for several months or even years [115,116]. During experimental studies, horizontal transmission was not observed after co-housing of uninfected cockatiels with experimentally PaBV-2- or PaBV-4-infected birds for at least five months [16,17,117]. Transmission of CnBV-1 in canaries was equally unsuccessful [17], whereas, in another study, at least two out of five canaries developed a clearly detectable CnBV-2 infection after contact with experimentally infected birds [19].

Oral uptake of contaminated feed or drinking water was assumed the most likely transmission route since infectious bornavirus was isolated from cloacal and pharyngeal swabs of infected birds, but, so far, this hypothesis lacks experimental confirmation [18,19]. When avian bornaviruses were experimentally inoculated parenterally by intramuscular, subcutaneous, intravenous or intracerebral injection using doses of approximately 10^3^ to 10^5^ focus-forming units (ffu) per bird, nearly all birds developed persistent infection. This was demonstrated for psittacines (mainly cockatiels) inoculated with PaBV-2 or PaBV-4 and for canaries inoculated with CnBV-1 or CnBV-2 [13,14,15,16,17,19,117,118,119,120,121,122,123,124]. Heckmann et al. [125] suggested that PaBV-4 infection may be established also by experimental inoculation via epidermal lesions. In contrast, mucosal inoculation of cockatiels with similar doses of PaBV-2 or PaBV-4 via peroral, intranasal or oculonasal routes did not result in detectable persistent infection, even when mucosal lesions had been induced before inoculation [119,125,126]. Oculonasal inoculation of canaries with high doses of CnBV-2 produced highly variable results in three different experiments, ranging from no or barely detectable infection to efficient infection of all inoculated birds [19,118]. Virus- or host-related factors, such as infectious dose and age, genetic background or immune status of the host, are speculated to influence the efficiency of transmission [118,126].

### 5.2. Vertical Transmission

While the epidemiological patterns of many avian bornaviruses cannot be explained by vertical transmission alone, they do not exclude the possibility of vertical transmission either [21]. In several studies, bornavirus RNA was detected in embryonated eggs originating from infected psittacines [127,128,129], canaries [19] or Canada geese [130]. Viral RNA in eggs was found to be correlated with viral shedding and wide tissue distribution in the organism of the mother but not with the father´s infection status [17,19]. Confirmation of a productive bornavirus infection of the embryos by detection of viral antigen in tissue slices or by virus isolation was not achieved [19,127]. Monaco et al. [129] detected bornavirus RNA in a cloacal swab from one out of ten artificially incubated and hand-reared chicks at five weeks of age. However, exposure to the virus post hatching could not be excluded completely since the chicks were kept within a facility also harboring infected adult birds. Thus, final proof of naturally occurring vertical transmission of avian bornaviruses is still missing.

### 5.3. Course of Infection

Depending on the infected hosts, cell and tissue tropisms of bornaviruses may differ markedly. Before the discovery of its natural reservoir, the mammalian BoDV-1 has long been considered as a strictly neurotropic virus that is found predominantly in the central nervous system. This assumption was based mainly on data from experimental models and naturally infected erroneous hosts [131,132,133,134]. In experimentally infected rats, rabbits and mice, the virus was demonstrated to spread mainly through intra-axonal transport along peripheral nerves. After retrograde infection of the central nervous system and subsequent replication in the brain, BoDV-1 may further disseminate centrifugally into peripheral organs [135,136,137,138]. In experimental hosts, such centrifugal dissemination is predominantly observed in the absence of a competent immune response, such as in immunocompromised animals or neonatally infected rats [132,133,138,139]. Despite this broader tissue distribution, the virus still remains largely bound to neuronal cells in these animals [132]. In consistence, viral shedding is usually not observed in erroneous and experimental non-reservoir hosts, although infectious virus was detected in the urine of neonatally infected rats [135,140].

Meanwhile, orthobornaviruses have been demonstrated to infect a broad range of tissues and cells in immunocompetent individuals of their respectively assumed reservoir hosts, such as BoDV-1 in bicolored white-toothed shrews [141,142,143], VSBV-1 in squirrels [36,144], canary bornaviruses in common canaries [19] and parrot bornaviruses in psittacines [86,89,122,145,146,147]. Cell types confirmed to be permissive for bornavirus infection in vivo included neurons, astroglia, ependymal cells, epithelial cells, smooth and skeletal muscle cells, myocardium and keratinocytes, demonstrating that orthobornaviruses are not, per se, strictly neurotropic [86,89,122,141,142,143,145,146,147,148,149,150].

In congruence with the broad tissue distribution and the infection of epithelial cells, viral shedding is a regular feature of orthobornavirus infections in their assumed reservoir hosts, and infectious virus has been detected in cloacal and pharyngeal swabs, saliva, feces and urine [16,18,19,143,144]. In experimentally infected birds, permanent shedding of high amounts of viral RNA usually starts after approximately eight to twelve weeks and is strictly correlated with an extensive tissue distribution. However, some birds do not have viral RNA detectable in their peripheral organs and excretions for at least several months. Nevertheless, high viral loads may be detectable in the brains of such birds [13,17,19,118,119,122,151].

Since only little data is available on tissue distribution early after infection, the mode of bornavirus dissemination in the organism of infected reservoir hosts is still a matter of debate. A strictly neuronal spread comparable to that of BoDV-1 in experimental non-reservoir hosts seems questionable given the broad range of infected cell types in these hosts. In line with this assumption, a single canary euthanized at two weeks after parenteral CnBV-1 injection was found to possess detectable viral RNA in all tested organs with the exception of the brain [17]. Furthermore, combined intramuscular and subcutaneous inoculation of cockatiels with PaBV-2 or canaries with CnBV-1 and CnBV-2 resulted in low amounts of viral RNA detectable in cloacal swabs of some of the experimentally infected birds as early as one week after inoculation. In most of these birds, virus shedding temporarily ceases until three to five weeks after inoculation [17,19,118,119]. Together, these observations suggest an initial systemic distribution during an early phase of the infection, indicating a mode of viral spread that is not exclusively neuronal. Strikingly, Leal de Araujo et al. [122] detected neither early dissemination of the virus nor detection of viral RNA in cloacal swabs earlier than five weeks after intramuscular PaBV-2 inoculation of cockatiels. Instead, their results suggested the virus to spread centripetally from the inoculation site in the pectoral muscle via the brachial plexus and the spinal cord to the brain, followed by centrifugal neuronal dissemination into other tissues. In such secondary replication sites, the virus also infected various non-neuronal cell types [122,150]. Early shedding is also not observed after experimental PaBV-4 infection of cockatiels and African grey parrots [17,118,151,152]. The discrepancies between the studies of different groups are not easily explained. Different virus isolates, dosages and inoculation routes as well as variable sensitivity of the assays used for virus detection may have contributed to the variations.

## 6. Clinical Signs, Gross Lesions and Histopathology

### 6.1. PaBV-Induced Disease in Psittacines

PDD and other bornavirus-induced diseases occur mainly in psittacines infected with parrot bornaviruses. The roles of PaBV-2 and PaBV-4 as causative agents of these disorders have been confirmed in numerous experimental infection studies in cockatiels [14,15,16,17,117,118,119,122,123], Patagonian conures [13] and African grey parrots [151]. Although final evidence is missing, it is widely assumed that further parrot bornaviruses, including the genetically more distantly related PaBV-5 and PaBV-6, are likewise pathogenic for psittacines.

**Figure 3:**
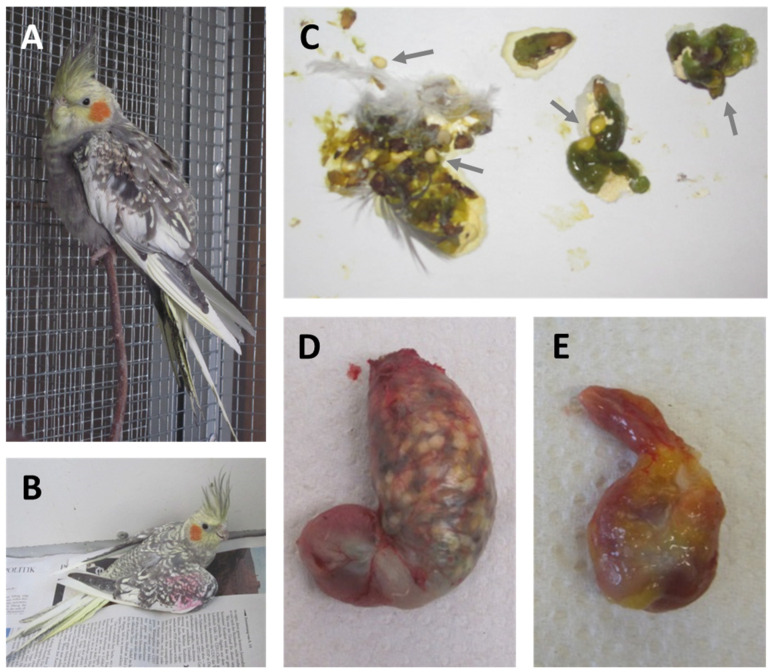
Clinical signs and gross pathology in experimentally PaBV-infected cockatiels. (**A**) Apathy and (**B**) lameness of an acutely diseased PaBV-4-infected cockatiel. (**C**) Shedding of undigested seeds (black arrow) in the feces of a PaBV-4-infected bird. (**D**) Gizzard and severely dilated proventriculus of a PaBV-2-infected cockatiel. Seeds are visible through the stretched and translucid proventricular wall. (**E**) Gizzard and proventriculus of a healthy, non-infected control bird. Adapted with permission from Refs. [17,119]. Copyright 2014, 2016, Elsevier.

Bornavirus-induced diseases in psittacines cover a considerable range of different clinical manifestations, with PDD being the most characteristic form. Typical PDD-like gastro-intestinal signs are proventricular dilatation (Figure 3D), delayed passage of ingesta, shedding of undigested seeds (Figure 3C) and diarrhea. As a result of the impaired digestion, birds often show body weight loss and emaciation [4,5,17,117,119,122,153]. In severe cases of proventricular dilatation, the proventriculus may rupture, resulting in peritonitis [4,5,153]. Neurological disorders represent an additional manifestation of avian bornavirus infections. Symptoms may include incoordination, seizures, tremors and lameness (Figure 3A,B) [4,15,17,151,154]. In addition, retinitis and blindness have been suggested as potential outcomes of avian bornavirus infections [155]. Behavioral disorders, such as feather plucking and auto-mutilation, have been described in association with avian bornavirus infections, but their causative relation requires further confirmation [84,151,154,156,157]. The course of disease is highly variable, ranging from peracute to chronic progression. Death without any prior clinical signs does occur in some birds [16,122]. However, the majority of birds die after chronic progression of the disease, whereas complete recovery is rarely reported [4,151,158]. The incubation period can be highly variable, ranging from three weeks to more than nine months in experimental studies, and a considerable proportion of infected birds may stay clinically healthy for several months or years or even become life-long healthy carriers [14,16,17,115,116,117,118,119,121,123,151].

Typical gross lesions of PDD are a dilated proventriculus with a thin and often transparent wall, whereas prominent macroscopic lesions in other organs are rare [16,17,117,119,121,123,145]. Microscopic lesions in the central nervous system are characterized by non-suppurative encephalitis, including mononuclear perivascular cuffing and focal gliosis. Neuritis and ganglioneuritis with mononuclear infiltrations can be observed in peripheral nerves as well as in neuronal ganglia of a broad range of organs [13,25,118,119,121,122,145,146,148,150,159]. Inflammatory cells in bornavirus-associated lesions were identified as mainly CD3-positive T lymphocytes and Iba1-positive macrophages/microglia in perivascular cuffings in the brains of experimentally PaBV-2-infected cockatiels, whereas infiltrates in ganglia of proventriculus and intestine were composed of T lymphocytes, macrophages as well as PAX5-positive B lymphocytes [160].

### 6.2. Bornavirus-Induced Disease in Passerines and Aquatic Birds

The pathogenic potential of the non-psittacine avian bornaviruses known to date is a matter of controversial debate. PDD-like disease, neurological disorders and typical mononuclear infiltrations have been described for domestic canaries naturally infected with canary bornaviruses [8,19,26]. However, experimental infection of canaries with CnBV-1 and CnBV-2 did not result in clinical disease, and only minimal histopathologic alterations were observed [17,19,118]. Naturally ABBV-1-infected Canada geese as well as gulls infected with an ‘ABBV-1-like’ virus have been described to suffer from neurologic diseases and exhibit mononuclear infiltrations in the brain, but suitable bornavirus-negative controls to demonstrate an association of virus and disease were not included in these studies [23,97,101,103,161]. Experimental infections reproducing ABBV-1-induced disease have not been performed yet. Likewise, no information is available on the pathogenicity of EsBV-1 and MuBV-1 in estrildid finches or ABBV-2 in ducks [19,20,22].

### 6.3. Avian-Bornavirus-Induced Diseases in Supposed Non-Reservoir Hosts

Bornavirus-related diseases in avian hosts other than their presumed primary hosts have been described for natural PaBV-4 infection of a Himalayan monal [94] and a natural ABBV-1 infection of an emu [104]. In both cases, neurologic disease and non-purulent encephalitis have been described, resembling diseases caused by BoDV-1 and VSBV-1 in erroneous mammalian hosts. These two mammalian orthobornaviruses establish persistent infections without causing disease in their known reservoir hosts, namely bicolored white-toothed shrews or exotic squirrels, respectively [105,149]. However, following transmission to non-reservoir hosts, such as domestic mammals or humans, they induce usually fatal neurologic disorders due to non-purulent encephalitis [105,162,163]. Further research is required to better understand the virus–host interactions of avian bornaviruses under spill-over conditions.

## 7. Pathogenesis of Bornavirus-Induced Diseases

Although considerable progress has been made over the past few years, comparably little information is available on the disease mechanisms in avian-bornavirus-infected birds. In contrast, the pathogenesis of BD has been extensively studied in BoDV-1 infection models in rats and mice [162]. Based on the close evolutionary relationship of avian and mammalian bornaviruses and on similarities of microscopic lesions, it is assumed that the pathogenesis of bornaviral diseases in birds may resemble that of Borna disease in BoDV-1-infected mammals [158]. Hence, this review provides a detailed summary of the published work on BoDV-1-induced immunopathogenesis and a discussion of the currently available information on avian bornaviruses.

### 7.1. Borna Disease in Mammals

Bornaviruses are known to establish non-cytolytic infections of their target cells, and direct damage is thus assumed to be limited [164,165]. Instead, BoDV-1-induced disease in non-reservoir hosts was shown to result from immunopathology mediated by virus-specific T lymphocytes [162].

The first evidence of an immunopathogenesis was achieved by observations that immunodeficiency in rats due to athymy, thymectomy or immunosuppressive treatment with cyclophosphamide, cyclosporine A (CsA) or corticosteroids resulted in dampened or even completely absent clinical disease and inflammatory infiltrates despite persistent BoDV-1 infection [139,166,167,168,169,170,171]. Adoptive transfer of splenocytes or brain lymphocytes derived from diseased immunocompetent BoDV-1-infected rats rapidly induced disease in infected immunocompromised healthy animals [166,169,171,172,173,174,175]. In contrast, transfer of reconvalescent sera containing virus-neutralizing antibodies did not induce immunopathology [172]. More detailed analyses of the roles of different lymphocyte subpopulations demonstrated that depletion or genetic knock-out of CD8+ T lymphocytes efficiently reduced or completely prevented BoDV-1-induced immunopathology in rats and mice, whereas less prominent effects were achieved by CD4+ T lymphocyte depletion [176,177,178,179,180]. In agreement, adoptive transfer of BoDV-1-specific CD8+ T cells was sufficient to induce immunopathology in persistently infected healthy individuals, whereas immunopathogenesis induced by the transfer of virus-specific CD4+ T cells appears to depend on the presence of CD8+ T lymphocytes [167,171,181,182,183]. Immunity against the bornavirus N protein appears to play a dominant role in the immunopathogenesis since immunization of N-encoding viral vector vaccines as well as transfer of N-specific CD8+ T lymphocytes or dendritic cells loaded with a BoDV-1 N epitope induced disease in persistently infected healthy mice [176,183,184,185]. Furthermore, transgenic N protein expression in neurons or astrocytes of mice prevented immunopathology following BoDV-1 infection, presumably due to immunotolerance [186]. Adoptive transfer of P-specific CD4+ T cells to infected rats likewise triggered immunopathology [171], whereas viral vectors encoding for P or G protein did not induce disease [176]. The precise mechanism of T-cell-mediated damage remains unknown. BoDV-1-infected mice lacking perforin, Fas, interferon γ (IFN-γ) or nitric oxide (NO) synthase developed disease indistinguishable from wild-type mice [187,188]. Stitz et al. [189] reported the treatment of rats with transforming growth factor β (TGF-β) to reduce inflammatory lesions in BoDV-1-infected rats.

The development of immunopathogenesis and disease in infected rodents is dependent on the age and genetic background of the host as well as on viral factors. Immune-mediated disease occurs in adult Lewis rats as well as in neonatally infected MRL mice, whereas black-hooded rats and most BALB/c or C57BL/6 mice remain healthy [169,177,190]. In mice, susceptibility to infection and immunopathology was shown to correlate with the major histocompatibility complex (MHC) haplotypes [177], whereas, in rats, no such association was observed [190]. In contrast to immunocompetent adult rats, neonatally infected rats usually do not develop typical neurologic disease and mononuclear infiltrates are absent from their brains, suggesting an age-dependent immunotolerance towards the virus [169,172]. However, some strains, such as Lewis, Fisher or Wistar rats, may exhibit unspecific clinical signs following neonatal BoDV-1 infection, with mild transient lymphocytic infiltration of the cortex but severe lesions and gliosis, particularly in the dentate gyrus [136,191,192,193]. Since these lesions are also observed in ex-vivo-infected hippocampal slice cultures, they are apparently independent of a T-cell-mediated immunopathology [191]. The dentate gyrus lesions are not developed by some rat strains, including Sprague Dawley or Brown Norway rats, which is attributed to a protective effect provided by an unidentified soluble factor produced in the brains of these rats [191]. Poenisch et al. [79] suggested X-protein-mediated anti-apoptotic effects to be required for preventing dentate gyrus lesions in neonatally infected Lewis rats. Subclinical deficiencies, such as impaired learning and memory functions, have been described for neonatally infected rats even in the absence of clinical signs and inflammatory lesions [194,195]. Host adaptation by multiple passages in experimentally infected hosts has been shown to increase infectivity and pathogenicity of the virus for rats and mice of all age groups [172,196,197,198,199].

T-cell-mediated immunopathogenesis is assumed to also mediate encephalitis in naturally BoDV-1-infected erroneous hosts, such as horses, sheep, humans and other mammals, although direct evidence is missing. By which mechanisms BoDV-1 is able to evade immunity and avoid immunopathology in its natural reservoir host, the bicolored white-toothed shrew, remains unknown.

### 7.2. PDD and Other Bornavirus-Induced Diseases in Birds

Most information available on the pathogenesis of avian bornavirus infections originates from captive psittacines. In these birds, clinical signs appear to be determined by the sites of bornavirus replication and subsequent development of encephalitis and/or ganglioneuritis [119,122,158]. Due to the usually broad tissue distribution, bornavirus-infected birds may develop central neurological signs as well as signs related to various internal organ systems, particularly the gastrointestinal tract. The precise mechanism leading to gastrointestinal dysfunction is largely unknown. Impaired gastrointestinal motility is assumed to be responsible for delayed transport of the ingesta, resulting in accumulation of feed in the dilated proventriculus, incomplete digestion and shedding of undigested seeds with the feces [158,200]. Peripheral ganglioneuritis may likewise disturb the function of other organ systems, such as the cardiovascular system, but the associated clinical signs have been characterized less well [159].

A recent study demonstrated that T-cell-suppressive treatment with CsA prevented inflammatory lesions and development of PDD in experimentally PaBV-2-infected cockatiels, suggesting a T-lymphocyte-mediated immunopathogenesis resembling that of BD [123]. Tools to determine the individual contributions of different T lymphocyte subpopulations are presently not available for psittacines. Hameed et al. [123] speculate that triggering a predominantly T_H_2-mediated immune response may prevent immunopathogenesis based on the observation that cockatiels immunized with alum-adjuvanted recombinant PaBV-4 N protein developed markedly reduced clinical disease and inflammatory lesions upon challenge infection with PaBV-2.

The range of possible outcomes in infected psittacines covers sudden death, acute to chronic fatal disease, recovery from disease as well as life-long healthy carrier status [4,16,17,115,117,118,119,122,158]. The conditions determining the fate of each infected individual are largely unknown. Recent work of Gartner et al. [124] demonstrated that, similar to BoDV-1 infection, age at the time of infection may be a decisive factor for the development of immunopathogenesis in PaBV-4-infected cockatiels. While nine out of eleven experimentally infected adults showed PDD-like disease, nestlings inoculated at the age of one to six days became healthy carriers, exhibiting inflammatory lesions without clinical disease [124]. In contrast to work with BoDV-1 in mammals [176], attempts to experimentally induce or exacerbate the disease in subclinically PaBV-4-infected cockatiels by subsequent immunization with viral vector vaccines expressing PaBV-4 N and P genes failed so far [152].

Based on parallels to mammalian bornavirus infections, multiple additional factors directly or indirectly influencing the antiviral immune response are assumed to play a role, including the genetic composition of virus and host, the host´s immune status at exposure to the virus, co-infections with other pathogens or stress. Furthermore, the degree of adaptation of the virus to the host species may be decisive. For instance, reproduction of disease in canaries by experimental infection with CnBV-1 or CnBV-2 failed completely [17,19,118], possibly reflecting a situation similar to non-pathogenic BoDV-1 infection in its shrew reservoir.

Autoreactive antibodies directed against gangliosides have been hypothesized as an alternative mechanism of the pathogenesis of PDD and ganglioneuritis in birds. However, evidence for the association of such antibodies with PDD-like lesions and clinical disease could not be presented so far [201,202,203].

## 8. Immunity against Bornavirus Infections

Immunity against bornaviruses has been studied most extensively for experimental BoDV-1 infection of rodents. This work demonstrated that—similar to their immunopathogenesis—antiviral protection is mediated mainly by T lymphocytes. Viral vector vaccines expressing the BoDV-1 N gene protected immunocompetent mice and rats against BoDV-1 challenge infection and development of BD [204,205], whereas protection was not achieved if the vaccinated mice lacked functional CD8+ T lymphocytes [204,206]. The important role of T lymphocytes received further confirmation by protective effects achieved by BoDV-1-specific T cells adoptively transferred to immunologically naïve rats prior to BoDV-1 challenge infection [182,207]. In contrast, humoral immunity appears to play only a minor role. Neither vaccination with inactivated virus nor purified viral antigen nor passive immunization with reconvalescent sera provided protective effects against BoDV-1 infection of rats [136,208,209]. Pre-treatment of rats with monoclonal antibodies directed against the G protein or reconvalescent sera possessing neutralizing activity did not affect BoDV-1 infection of the brain but was able to delay viral spread from or to the periphery and prevent viral shedding in the newborn rat BoDV-1 infection model [132,210]. In experimentally infected animals, neutralizing antibodies become detectable only after several weeks of persistent infection, and their appearance is not associated with viral clearance [65,210,211].

Similar data for avian bornavirus infections are scarce so far. Detection of specific T cells in exotic bird species is, at present, impossible due to the lack of suitable tools and methods. Thus, investigation of immune responses is restricted mainly to measuring bornavirus-reactive antibodies during persistent infection. Antibodies detected in infected birds are predominantly directed against N, P, X and M proteins, whereas antibodies directed against G and L or neutralizing antibodies have not been detected [212]. Whether these antibodies contribute to controlling the infection remains questionable since high titers of bornavirus-reactive antibodies are regularly detected in naturally and experimentally bornavirus-infected birds regardless of their infection status or development of disease [16,17,83,117,118,119].

## 9. Diagnosis of Avian Bornavirus Infections

The diagnosis of avian bornavirus infections is based on both direct and indirect virus detection. A major challenge is the considerable genetic variability of this virus group. Furthermore, persistently infected birds do not always shed the virus or develop a specific antibody response. Therefore, a combination of direct virus detection and serology is recommended for intra vitam diagnosis.

### 9.1. Direct Avian Bornavirus Detection

Avian bornavirus detection is mainly based on viral RNA detection by reverse transcription-polymerase chain reaction (RT-PCR) methods. Among the samples collected intra vitam, cloacal swabs usually contain higher amounts of viral RNA as compared to pharyngeal swabs and whole blood samples [18,122]. In addition, viral RNA is also detectable in urine samples [147] and feather calamy [212]. Post mortem, the highest loads of viral RNA and antigen are usually detected in organs rich in neuronal tissue, such as brain, eye and adrenal gland, whereas low amounts of virus are often found in liver, spleen and skeletal muscle [16,17,117,118,122,213]. However, depending on the course of infection, not all samples are reliably positive in all persistently infected birds. In the majority of infected birds, the virus is widely distributed in all organs and readily detectable in their excretions. However, some individuals possess high viral loads only in the central nervous system, while the virus is barely detectable in other organs and swab samples [16,17,19,115,117,118,119,122,146]. In rare cases, low viral loads are detected only in a few organs but not in the central nervous system, presumably due to an early phase of infection or permanent restriction of viral spread by the immune system [17,118,119,122].

#### 9.1.1. Detection of Avian Bornavirus RNA

Nowadays, (semi-)quantitative TaqMan-based real-time RT-PCR assays (RT-qPCR) are becoming the standard assays for direct avian bornavirus detection due to their high sensitivity and specificity, reduced hands-on time and lower risk of laboratory cross-contamination as compared to conventional RT-PCR assays. However, several aspects have to be considered for reliable RT-qPCR-based avian bornavirus detection, such as the considerable genetic diversity of avian bornaviruses. As their nucleotide sequences may differ by more than 30% [27], most RT-qPCR assays are able to detect only one particular avian bornavirus or few closely related viruses [214].

RT-qPCRs predominantly detecting one particular avian bornavirus have been described for PaBV-2 [117,119], PaBV-3, PaBV-4 [1], CnBV-2 [118] and ABBV-1 [97,214]. Additional RT-qPCRs have been designed for the detection of the members of either the species *Orthobornavirus alphapsittaciforme* (PaBV-1, to -4, -7, -8) [213,214,215] or *Orthobornavirus avisaquaticae* (ABBV-1 and -2) [102,214]. In addition, Sigrist et al. [214] established a multiplex RT-qPCR for the simultaneous detection of the members of the species *Orthobornavirus alphapsittaciforme* (PaBV-1, to -4, -7, -8), *Orthobornavirus serini* (CnBV-1 to -3, MuBV-1), *Orthobornavirus estrildidae* (EsBV-1) and *Orthobornavirus avisaquaticae* (ABBV-1 and -2). Only one published RT-qPCR assay, named panBorna 7.2 [216], was demonstrated to detect a broad range of avian and mammalian orthobornaviruses [214]. In addition, several conventional PCR assays have been confirmed to not only detect a broad spectrum of known orthobornaviruses but also helped to discover previously unknown members of the genus *Orthobornavirus* [2,18,19,20,24,25,26,214].

However, the broadly reactive are usually less sensitive as compared to virus- or species-specific RT-qPCR assays [214]. Thus, an educated decision on the use of assays is crucial for a reliable diagnosis based on profound knowledge on the avian bornaviruses expected in the sampled population and on the characteristics of the assays of choice. For instance, PaBV-4 is the most widely distributed bornavirus of parrots [21,25], justifying the use of a PaBV-4-specific RT-qPCR assay [1] for PaBV-4 detection from parrot samples with maximal sensitivity. Since this assay does not detect or only very inefficiently detects any other avian bornavirus, it needs to be combined with assays able to detect the further known psittacine bornaviruses PaBV-1 to -3, -7 and -8 (*Orthobornavirus alphapsittaciforme*) and PaBV-5 and -6 (*Orthobornavirus betapsittaciforme*). For samples with an undefined spectrum of expected bornaviruses, the use of more than one broad-spectrum bornavirus RT-PCR is recommended to minimize the risk of missing virus [19,20,214].

In addition to PCR, viral nucleic acids can also be visualized in tissue slices by in situ hybridization (ISH) using probes specific for PaBV-2 or PaBV-4 [89].

#### 9.1.2. Detection of Avian Bornavirus Antigens

Bornavirus antigens can be detected by immunofluorescence assays (IFA) and immunohistochemistry (IHC) performed on tissue slices [16,25,83,86,89,145,146,148,213,217] or by Western blot (WB) or antigen capture ELISA from homogenized organ samples, swabs as well as from feather calamy [218,219]. Polyclonal rabbit sera directed against various antigens of BoDV-1 or avian bornaviruses have been shown to readily react with antigens of a broad range of avian bornaviruses [25,75,86,146,148,217,219]. Due to this high degree of serological cross-reactivity within the genus *Orthobornavirus*, antigen detection assays are much less prone to produce false negative results caused by genetic and antigenic variability [75]. However, antigen detection is usually markedly less sensitive as compared to RT-PCR [17,19,213].

#### 9.1.3. Avian Bornavirus Isolation

Isolation of infectious virus is possible from organ samples [13,18,19,20,23,86,217] as well as from samples collected intra vitam, such as cloacal and pharyngeal swabs and blood samples [18,19]. A broad range of avian cell lines may be used for this purpose, including CEC-32 quail fibroblasts, QM7 quail muscle cells, QT6 and QT35 quail fibroblasts, DF-1 chicken fibroblasts, CCL-141 duck fibroblasts and primary duck embryo fibroblasts [13,18,19,20,23,86,217,220,221,222]. In contrast, avian bornaviruses replicate only inefficiently in mammalian cells [18,19,20,86]. Bornaviruses are slowly replicating viruses that may take several weeks to successfully establish persistent infection of the complete culture. Due to the absence of a bornavirus-induced cytopathic effect, IFA, immuneperoxidase staining (IPO) or RT-PCR are required to confirm successful virus isolation [18,19,20,86,222]. Bornavirus isolation is time-consuming and laborious and, thus, not widely used for routine diagnosis. However, it represents a valuable tool for scientific studies.

### 9.2. Indirect Detection of Avian Bornavirus Infections

Since some bornavirus-infected birds are not or only intermittently shedding viral RNA, serology is an important parameter of intra vitam diagnosis [83,115]. In principle, unequivocally confirmed seroconversion can be considered as evidence of bornavirus infection as avian bornaviruses establish lifelong persistence and vaccines are not available. However, bornavirus serology has been discussed to be prone to false positive results in the past [223]. Furthermore, not all bornavirus-infected birds develop detectable bornavirus-reactive antibodies [19,83,115]. Thus, serology should always be combined with direct virus detection.

Immunofluorescence antibody assay (IFAT; synonym indirect immunofluorescence assay, iIFA), enzyme-linked immunosorbent assays (ELISA) and WB assays are used for the detection of bornavirus-specific antibodies [75,91,116,128,212,217,218,224]. Cells persistently infected with various bornaviruses can be employed for antibody detection by IFAT [75,217]. ELISAs using recombinant proteins produced in *Escherichia coli* have been designed to detect antibodies reacting with individual bornavirus antigens, such as N, P, X or M protein [19,116,128,212]. Detection of specific antibodies by WB assays can be performed with either recombinant proteins or bornavirus antigen originating from persistently infected cells [91]. Despite the considerable serologic cross-reactivity, the use of a heterologous bornavirus antigen for the detection of antibodies directed against a genetically divergent bornavirus may considerably reduce the measured antibody titers as well as the sensitivity of the assay [75]. Therefore, a purposive choice of the antigen source according to the expected range of bornaviruses in the sampled population is crucial for reliable serological diagnosis.

### 9.3. Clinical and Pathological Diagnosis of Bornavirus-Induced Disease in Birds

Since not all bornavirus-infected birds develop clinical disease, evidence of an avian bornavirus infection is not equivalent with the diagnosis of PDD [158]. Symptoms typical of PDD are gastrointestinal signs, such as shedding of indigested seeds, a dilated proventriculus and a delayed gastrointestinal passage of the ingesta, visualized by contrast radiographic methods [5]. Furthermore, bornavirus-induced disease may also include various neurologic disorders as well as non-specific symptoms, such as chronic emaciation, apathy, ruffled feathers or sudden death without previous signs of disease [4,5]. While typical gastrointestinal signs and, to some extent, neurologic disorders are suggestive of PDD, none of these conditions are pathognomonic for the disease. Similar clinical manifestations can be caused by a broad range of other infectious and non-infectious agents, such as *Macrorhabdus ornithogaster*, endoparasites, heavy metal intoxications, neoplasia or other neurotropic pathogens [4,5,200]. Confirmation of the diagnosis of PDD is mainly achieved by microscopic detection of mononuclear infiltrations in the central nervous system and peripheral ganglia [5]. Crop biopsies have been used for microscopic analysis intra vitam, but ganglioneuritis is not always detectable in these tissues even in birds affected by PDD [12]. Alterations in the hematological parameters clearly associated with avian-bornavirus-induced disease, allowing a clinical diagnosis or facilitating prognosis, have not been identified [5,151].

## 10. Prophylaxis and Therapy of Bornavirus Infections and PDD

To date, an effective causative or clinical therapy does not exist and a specific immunoprophylaxis is not commercially available. Thus, the control of avian bornavirus infections and PDD is currently restricted mainly to flock management measures based on extensive monitoring, followed by separation of infected birds to eradicate the virus from the flock.

### 10.1. Antiviral Compounds

Antiviral compounds have been tested for inhibitory effects on bornavirus infection predominantly in cell culture, whereas confirmation in vivo is usually missing.

The nucleotide analogue ribavirin was shown to inhibit mammalian and avian bornaviruses in various cell lines. Proportions of virus-positive cells and viral RNA levels in persistently infected cell cultures were markedly reduced, and viral spread was blocked or at least delayed after de novo infection of cultures [225,226,227,228,229]. However, elimination of the viruses from persistently infected cultures was not achieved, and infection rapidly recovered after cessation of treatment [225,226,227,228,229,230]. The antiviral mechanisms of ribavirin against bornaviruses remain unknown. Increased mutation rates leading to an ‘error catastrophe’, as shown for other RNA viruses, were not detected by next generation sequencing [226]. Conflicting results were published regarding ribavirin-mediated reduction of the cellular guanosine triphosphate (GTP) pool as a potential mechanism of bornavirus inhibition [225,226,227]. Reuter et al. [226] showed a marked inhibition in a PaBV-4 polymerase reconstitution assay, suggesting a direct effect of ribavirin on the viral polymerase. Furthermore, ribavirin was shown to enhance type I IFN signaling in avian cells [226]. In vivo treatment of experimentally BoDV-1-infected rats or gerbils by repeated intracerebral ribavirin injections revealed a slight reduction in viral loads and neurological signs, but virus elimination was not achieved [231,232]. Oral ribavirin treatment of parrots was attempted but did either not achieve any effect on viral shedding [233] or even induced detrimental effects on the birds [226]. Recently, the antiviral compound favipiravir (T-705) was demonstrated to inhibit BoDV-1 and PaBV-4 in cell culture with markedly higher efficiency as compared to ribavirin and may be able to eliminate bornaviruses entirely from persistently infected cultures [230]. It remains to be elucidated whether favipiravir is able to provide an antiviral effect also in vivo and whether treated birds tolerate the drug.

Recombinant chicken IFN-α inhibited avian bornaviruses in quail cell lines, but, similar to ribavirin, complete elimination was not achieved [226,234]. A combination of ribavirin and IFN-α resulted in an enhanced antiviral effect, suggesting a synergistic action of both [226]. However, recombinant psittacine IFN-α was not available for the evaluation of the efficacy of in vivo IFN-α treatment.

Amantadine was suggested to inhibit BoDV-1 infection in cell culture [235], but several subsequent studies were not able to reproduce this antiviral effect [236,237,238]. Clinical improvements in human psychiatric patients who claimed to be BoDV-1-infected are likely a result of the extensively studied direct antidepressant effect of this compound rather than of an antiviral action [239,240]. The effect of amantadine against avian bornaviruses has not been tested so far.

### 10.2. Therapy of Proventricular Dilatation Disease

Treatment of PDD is mainly restricted to symptomatic measures due to the lack of a causative therapy. Several strategies have been proposed to improve conditions of PDD-affected birds, but reports are often conflicting and systematic studies are generally missing [4,5,202,233].

Based on the suspected T-cell-mediated pathogenesis, immunosuppressive drugs inhibiting T lymphocytes, such as CsA, are obvious candidates for treatment or prevention of PDD. Hameed et al. [123] reported that daily peroral treatment of experimentally PaBV-2-infected cockatiels with 0.2 mg CsA starting at the day of infection efficiently prevented the development of PDD despite persistent bornavirus infection. In contrast, evidence for a successful therapeutic use of CsA at a later stage of experimental or natural avian bornavirus infection is still missing. While some authors reported a beneficial effect, others observed no effect on lesion development but a wider organ distribution of the virus [4,233]. In addition, the use of other anti-inflammatory compounds, such as the cyclooxygenase 2 (COX-2) inhibitors celecoxib, robenacoxib or meloxicam, has been described [4,5,233]. In experimental studies, neither meloxicam nor celecoxib induced beneficial effects in PaBV-2- or PaBV-4-infected cockatiels [120,241]. Furthermore, gastrointestinal prokinetic agents, such as metoclopramide, have been suggested to provide beneficial effects during the early phase of disease [202].

### 10.3. Vaccination

To date, no vaccines for the protection against avian bornavirus infections are commercially available. Experimental studies using two different approaches have been reported, employing either recombinant bornavirus N protein [123] or viral vector vaccines expressing bornavirus N and P proteins [118,119,152].

Cockatiels immunized with alum-adjuvanted, *Escherichia coli*-expressed PaBV-4 N protein were not protected against challenge infection with PaBV-2, demonstrated by high levels of viral RNA detectable in tissues and cloacal swabs. Strikingly, only a minority of vaccinated birds developed PDD-associated microscopic lesions and clinical disease, suggesting that vaccination prevented immunopathology by modulating the immune system [123]. Despite clinical protection, this approach appears not suitable as a vaccination strategy due to its lack of protection against infection. Healthy, vaccinated life-long virus carriers shedding high amounts of virus would be Trojan horses that facilitate the spread of avian bornaviruses and obstruct efforts to eradicate these viruses from parrot populations.

In contrast, live viral vector vaccines expressing bornavirus antigens provided partial or complete protection against homologous and heterologous bornavirus infection of cockatiels and canaries and, consequently, also against PDD-like lesions [118,119,152]. In these studies, recombinant vectors based on Newcastle disease virus (NDV) clone 30, modified vaccinia virus Ankara (MVA) and orf virus (ORFV) were designed to carry the N and P genes of PaBV-4 or CnBV-2. They were demonstrated to be safe and to induce bornavirus-specific seroconversion immunogenic in cockatiels and canaries [118,119,152]. A heterologous prime-boost vaccination regime combining the NDV and MVA vector systems delayed the course of challenge infection with high doses of PaBV-4 or CnBV-2 in cockatiels or canaries, respectively. However, persistent infection was not prevented in most of the vaccinated birds, and two vaccinated cockatiels developed PDD [118]. In contrast, the same prime-boost regime using PaBV-4 vector vaccines provided almost complete protection against heterologous challenge with a 10-fold lower dose of PaBV-2. Minimal levels of viral RNA were detected in only two out of six vaccinated animals and none of the birds developed PDD-like clinical signs or histopathology [119]. Complete protection of cockatiels against PaBV-2 challenge infection and disease was achieved by three consecutive injections of PaBV-4-expressing MVA vaccines, but not by using the NDV or ORFV vectors [152].

These results indicate that a strong vector-vaccine-induced immune response, presumably mediated by specific T lymphocytes, is required to eliminate the virus completely or at least sequester the challenge infection permanently and thereby prevent the development of immunopathogenesis and disease [119,152], whereas incomplete protection against bornavirus infection does not hamper disease development but may even contribute to immunopathogenesis [118]. Similar observations had been made for vaccination of rats against BoDV-1 infection. Lewis et al. [242] observed reduced challenge virus titres accompanied by more pronounced disease progression in mice vaccinated with a vaccinia virus (VV) construct encoding BoDV-1 N.

### 10.4. Control of Avian Bornavirus Infection by Flock Management

In the absence of available vaccines and causative therapies, avian bornaviruses are controlled mainly by flock management. The ultimate goal of such measures is the eradication of the viruses from a flock and the prevention of its reintroduction. Eradication programs are based on repeated testing of all birds of swab samples by suitable RT-PCR methods combined with serology, followed by separation—and, finally, removal from the flock—of all detectably bornavirus-infected individuals. Newly introduced birds originating from flocks with unknown or potentially positive bornavirus status need to be kept in quarantine until repeated testing has confirmed the absence of avian bornavirus infection [4].

Flock monitoring and testing during quarantine are complicated by the fact that some infected animals may be negative for viral shedding and seroconversion, resulting in false negative results [115]. Thus, eradication programs may need to be enforced over several months or even years to ensure the bornavirus-free status of a flock. The unknown mechanisms of avian bornavirus transmission are a further obstacle for implementation of efficient control measures. However, the apparently inefficient transmissibility and the slow spread within populations provide a possibility for thoroughly performed control and prevention strategies to be successful [4]. Murray et al. [243] described a cockatiel flock initially harboring three confirmed PaBV-4-positive animals. Subsequent testing of the flock suggested that the virus was successfully eradicated after the death of these three birds.

## 11. Conclusions and Future Perspectives

Almost 15 years of active research have yielded ample knowledge on avian bornavirus infections and PDD. The considerable genetic and biological variability of this group of viruses is appreciated nowadays, and diagnostic tools are progressively implemented to cope with this fact. Important recent contributions have led to an incipient understanding of the pathogenesis of avian-bornavirus-induced diseases, revealing striking parallels to Borna disease in mammals. Experimental studies have provided a proof of concept for successful immunoprophylaxis against avian bornavirus infections using live viral vector vaccines.

However, many aspects of avian bornavirus infections and diseases remain elusive. The routes and mechanisms of natural bornavirus transmission are poorly understood, not only for avian bornaviruses but also for their mammalian relatives. Except for ABBV-1, the wild reservoirs of avian bornaviruses, their geographic origin and their way of introduction into captive bird populations are unknown. Likewise, the potential threat posed by their (re-)introduction into wild populations of endangered species remains largely speculative. Prophylactic measures are still restricted to quarantine, diagnostic monitoring and subsequent separation of infected birds since therapeutic approaches and vaccines are still in experimental phases and are unlikely to become available in practice soon. These and other issues provide manifold prospects for future avian bornavirus research. Finally, additional avian bornaviruses may be discovered, possibly belonging also to alternative bornavirus genera, such as *Carbovirus* or *Cultervirus*, potentially raising further open questions.

## Figures and Tables

**Table 1:** Taxonomy of the genus *Orthobornavirus*.

Species	Virus Name	Abbreviation	Abbreviation of Abandoned Name	Representative Sequence
*Orthobornavirus alphapsittaciforme*	parrot bornavirus 1	PaBV-1	ABV-1	JX065207
	parrot bornavirus 2	PaBV-2	ABV-2	EU781967
	parrot bornavirus 3	PaBV-3	ABV-3	FJ169440
	parrot bornavirus 4	PaBV-4	ABV-4	JX065209
	parrot bornavirus 7	PaBV-7	ABV-7	JX065210
	parrot bornavirus 8	PaBV-8	ABV-8	KJ950619
*Orthobornavirus betapsittaciforme*	parrot bornavirus 5	PaBV-5	ABV-5	LC120625
	parrot bornavirus 6	PaBV-6	ABV-6	FJ794726
*Orthobornavirus serini*	canary bornavirus 1	CnBV-1	ABV-C1	KC464471
	canary bornavirus 2	CnBV-2	ABV-C2	KC464478
	canary bornavirus 3	CnBV-3	ABV-C3	KC595273
	munia bornavirus 1	MuBV-1	ABV-LS	DC290659
*Orthobornavirus estrildidae*	estrildid finch bornavirus 1	EsBV-1	ABV-EF	KF680099
*Orthobornavirus avisaquaticae*	aquatic bird bornavirus 1	ABBV-1	ABV-CG	KF578398
	aquatic bird bornavirus 2	ABBV-2	ABV-MALL	KJ756399
*Orthobornavirus bornaense*	Borna disease virus 1	BoDV-1	BDV	U04608
	Borna disease virus 2	BoDV-2	BDV No/98	AJ311524
*Orthobornavirus sciruri*	variegated squirrel bornavirus 1	VSBV-1	-	LN713680
*Orthobornavirus caenophidiae*	Caribbean watersnake bornavirus	CWBV	-	BK014571
	Mexican black-tailed rattlesnake bornavirus	MRBV	-	BK014572
*Orthobornavirus elapsoideae*	Loveridge’s garter snake virus 1	LGSV-1	RBV-1	KM114265
not classified	Gabon viper virus 1 *	GaVV-1	RBV	AB714966

Virus names, abbreviations and species designations are listed according to the taxonomy and nomenclature approved by the International Committee on Taxonomy of Viruses (ICTV) [28]. Formerly used abbreviations of obsolete virus names are provided to allow for correlation of literature published before or after the taxonomic reorganization of the family *Bornaviridae* in 2015 [27]. * GaVV-1 has not yet been classified by the ICTV due to insufficient sequence information.

## Data Availability

Not applicable.
